# Inter-observer and Intra-observer Reproducibility for the Determination of Female Urethral Narrowing on Voiding Cystourethrogram: A Retrospective Study

**DOI:** 10.7759/cureus.101464

**Published:** 2026-01-13

**Authors:** Alea I Zone, Caitlin E Carlton, Mortadha Al-Kinani, Gaurav Khatri, Kristen Bishop, Theresa Huang, Alana L Christie, Philippe E Zimmern

**Affiliations:** 1 Department of Urology, University of Texas Southwestern Medical Center, Dallas, USA; 2 Department of Radiology, University of Texas Southwestern Medical Center, Dallas, USA

**Keywords:** urethral narrowing, urethral stricture, urogynecology, vcug, voiding cystourethrogram

## Abstract

Background

The voiding cystourethrogram (VCUG) is used in women to evaluate the anatomy of the lower urinary tract. Once common for detecting reflux, urethral mobility, and diverticula, its use in adults has declined. However, VCUG remains valuable in urogynecology and reconstructive pelvic surgery (URPS) by identifying structural causes of recurrent urinary tract infections (RUTI), such as urethral pathology.

Objective

The main objective of this study is to evaluate the inter and intra-observer variability among radiologist interpretations of urethral caliber and narrowing on VCUG.

Methods

Following IRB approval, 83 VCUG cases performed on non-neurogenic women between 18 and 85 years old, and without prolapse >stage 2, were identified retrospectively from a tertiary care urology clinic between January and July 2023. All studies were performed in the upright sagittal oblique projection. Each image was independently reviewed by four abdominal fellowship-trained radiologists, who judged whether each urethra was narrow or not. If narrow, the rater noted severity (mild, moderate, severe) and location (proximal, mid, distal). The Shrout-Fleiss reliability statistic for a fixed set of raters (intra-class correlation (ICC): 3, k) was then calculated for the presence or absence of narrowing.

Results

In 50/83 (60%) of cases, all raters agreed on whether the urethra was narrow. Majority agreement (at least three of four raters) was present in 75/83 (90%) of cases. The ICC (3, k) was 0.79 for the reliability of the four scores. For the 61 cases rated as narrow by at least three of the four radiologists, the majority agreed on the degree of severity in 43/61 (70%; ICC = 0.89) of cases, and on location in 51/61 (84%; ICC = 0.43) of cases.

Conclusion

To our knowledge, this is the first study to assess the inter-reader reproducibility of urethral narrowing on standing VCUG in women with lower urinary tract symptomatology. A majority agreement was reached on urethral narrowing, including its location and severity. Correlating VCUG findings with clinical data will help establish a standardized radiologic definition of urethral narrowing.

## Introduction

In the field of urogynecology and reconstructive pelvic surgery (URPS), the role of the voiding cystourethrogram (VCUG) in evaluating the bladder and urethra during filling and voiding has varied over the years. Historically, the VCUG has been used to detect reflux [1,2]; to objectively assess the degree of mobility of the urethra and bladder base in the standing position by comparing rest and straining views in women with urinary incontinence [3] and/or cystocele [2,4]; to identify urethral pathologies such as diverticulum or narrowing [5,6]; and to confirm a suspected vesico- or urethro-vaginal fistula, or its resolution after repair [7,8]. The VCUG has also been useful in men and women with voiding dysfunction to localize the site of a possible obstruction on lateral voiding views [6,9].

With the increase in antibiotic-recalcitrant recurrent urinary tract infections (RUTIs) in women, VCUG has at times been included to detect infrequent etiologies that may be overlooked, such as urethral diverticulum, reflux, or bladder diverticulum. VCUG is one of three modalities used to evaluate voiding in women, along with non-invasive flow studies or more formal urodynamic testing. VCUG is readily available at most centers and requires limited radiation exposure [1]. However, the interpretation of the VCUG voiding views remains challenging, given the relative infrequency of this examination in adults. Definitions of urethral narrowing in adult women on VCUG have not been formally established or reported in the literature, and there is a paucity of studies assessing radiologist interpretation of VCUGs in adults. Therefore, this study aims to determine the reliability of radiologists in reading female VCUGs, particularly in evaluating urethral narrowing and its exact location along the urethra. Radiologic reproducibility is crucial to URPS practice, as VCUG interpretation can significantly influence the diagnostic evaluation and clinical management of female voiding dysfunction.

## Materials and methods

Following IRB approval, a retrospective study was conducted at a tertiary urology clinic at an academic referral center between January and July 2023. VCUG studies ordered in adult women with a history of RUTIs were retrospectively reviewed. Patients with a diagnosis of neurogenic bladder or pelvic organ prolapse higher than stage 2 were excluded from this study. VCUG images were performed using a standardized institutional protocol in upright sagittal oblique projections. The protocol has been used for over two decades and includes a sequence of several views, including sagittal oblique and lateral views of the bladder at 125 mL, at rest and strain; at maximum capacity, at rest and strain; an anterior/posterior (AP) view of the bladder at capacity at rest; a sagittal oblique voiding view; and AP views of the upper and lower abdomen post-void [1].

Imaging review

**Figure 1:**
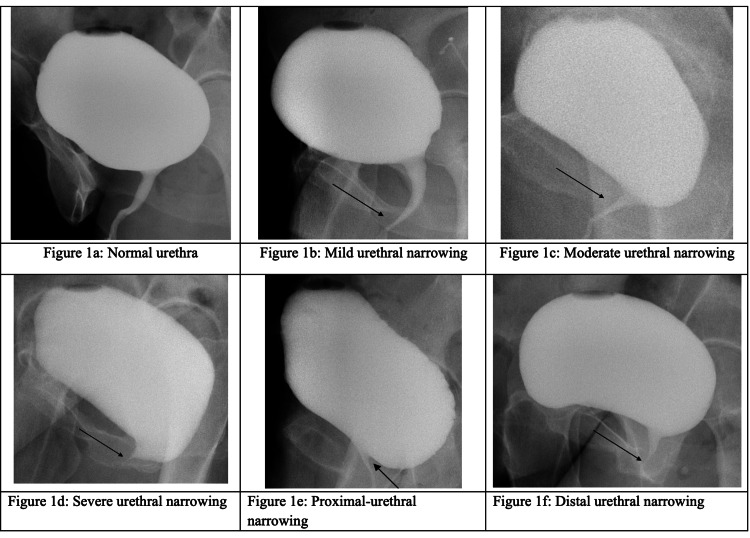
Representative images provided in a PowerPoint presentation (Microsoft® Corp., Redmond, WA, USA) used to train the four participating radiologists on VCUG reading protocol. VCUG, voiding cystourethrogram

Four fellowship-trained radiologists retrospectively reviewed each VCUG sequence independently. All reviewers were blinded to subject demographics and clinical histories. They accessed images through the Department of Radiology’s secure Picture Archiving and Communication System (PACS) and were able to review all images of each VCUG study. Before reviewing the VCUG cases, the radiologists conducted a consensus review of a training set of images that included multiple examples of normal urethra and urethra with proximal, mid, or distal narrowing, as agreed upon by the four radiologists (Figures 1a-1f). Normal urethras had an equal caliber throughout, while narrow urethras demonstrated some distention proximal to an apparent reduction in caliber or a narrow caliber along their entire length. During the independent, blinded review, the radiologists subjectively indicated the presence or absence of urethral narrowing. If narrowing was present, the radiologists graded the severity of the narrowing on a scale from 1 to 3 (mild, moderate, severe). The radiologists then classified the location of the narrowing as either proximal, mid, distal, or “other” (to include overlap in location, such as mid-to-distal or proximal-to-mid). Graders characterized urethral location using numerical estimation (i.e., 1 cm per segment for a total length of 3 cm) or functional parameters based on nearby anatomy [10]. This initial review took place in September 2023. For the inter-observer reliability study, each radiologist independently read 83 studies. For the intra-rater reliability study, the same four radiologists repeated their reads on 10 randomly selected studies from the original batch over a year later, in May 2025, to ensure no recollection bias. 

Statistical analysis

The primary outcome in this study was inter-observer agreement, or the number of reviewers who agreed on a particular read. Participating radiologists reported on the presence, degree, and location of urethral narrowing. There were four possible outcomes for each case: complete agreement (all reviewers agreed), majority agreement (three of four or two of three reviewers agreed), split call (reviewers divided equally between two opinions), and complete disagreement (all reviewers disagreed in the degree or location metrics). The Shrout-Fleiss intraclass correlation coefficient (ICC) was calculated to assess the degree of intra- and inter-observer reliability, with poor correlation defined as a score less than 0.4, fair correlation between 0.4 and 0.59, good correlation between 0.6 and 0.74, and excellent correlation at 0.75 or greater [11]. All analyses were performed using SAS 9.4 (SAS Institute Inc., Cary, NC, USA).

## Results

This study examined VCUG images from 83 women aged 18 to 85 years old. Regarding urethral narrowing, in 60% of images (50/83), all reviewers agreed on 44 narrow and 6 not narrow urethras (Table 1). Specifically, 90% of all images (75/83) demonstrated majority agreement for 61 narrow and 14 not narrow urethras. Conversely, 10% of images (8/83) demonstrated a split opinion. The ICC was 0.79 (95% confidence interval (CI): 0.71, 0.86), signifying high reliability for this metric across the four reviewers [11]. Reviewers were less likely to agree when measuring the degree of urethral narrowing. However, ICC was higher in part due to having more categories (ICC = 0.89; 95% CI: 0.84, 0.93), with raters tending to score within one level of each other (e.g., mild and moderate) rather than scoring on opposite ends of the range (e.g., mild and severe). For the 61 cases that most radiologists deemed narrow, all reviewers agreed on the severity in only 34% of cases (21/61): 13 mild, 1 moderate, and 7 severe. A majority agreement on severity was reached in 70% of these cases (43/61): 21 mild, 11 moderate, and 11 severe. The remaining 30% of cases (18/61) were split calls between two severity ratings.

**Table 1:** Reviewer agreement on lateral view of VCUG readings. The majority agreement includes all cases with complete agreement. VCUG, voiding cystourethrogram; ICC, intra-class correlation

Urethral characteristics	Complete agreement	Majority agreement	No consensus	ICC (95% CI)
Presence of narrowing (n = 83)	50 (60%)	75 (90%)	8 (10%)	0.79 (0.71, 0.86)
Severity of narrowing (n = 61)	21 (34%)	43 (70%)	18 (30%)	0.89 (0.84, 0.93)
Location of narrowing (n = 61)	28 (46%)	51 (84%)	10 (16%)	0.43 (0.13, 0.64)

One of the four reviewers categorized location as “other” in 71% of their image reads, including 22 called “diffuse” and 24 with two locations listed. If we exclude the reader who primarily used “other” to categorize location, we find that, in the 61 cases that most radiologists deemed narrow, the three reviewers agreed on the location of narrowing in 46% of cases (28/61; ICC = 0.43; 95% CI: 0.13, 0.64): 6 proximal, 16 mid, and 6 distal. A majority agreement on severity was reached in 84% of these cases (51/61): 11 proximal, 31 mid, and 9 distal. The remaining 16% of cases (10/61) were full disagreements.

Regarding intra-rater reliability on 10 repeat reads, one rater agreed on 7 reads, two raters agreed on 9 reads, and the final rater agreed on all 10 reads.

## Discussion

This single-institution, retrospective study on interpreting the presence or absence of urethral narrowing in the voiding phase of VCUG studies in adult women, conducted by four fellowship-trained radiologists, demonstrated high inter- and intra-observer agreement. Additionally, there was significant agreement regarding the severity and location of urethral narrowing. 

There is currently no standardized definition for urethral narrowing, nor are there guidelines for grading the severity of narrowing based on VCUG results. In its 2023 Urethral Stricture Guideline Amendment, the American Urological Association (AUA) designates ultrasound urethrography as the primary method for assessing the severity and location of narrowing in males [12]. Urethrography is both sensitive and specific for anterior narrowing, but it is limited by patient discomfort and the ultrasonographer's skill [12]. The AUA guidelines suggest that VCUG may be helpful in assessing female narrowing [12]. Similarly, the American College of Radiology (ACR) recommends VCUG as the preferred modality for assessing urinary dysfunction, as it allows for physiologic upright positioning, voiding assessment, and full bladder visualization [13].

Both groups emphasized the utility of VCUG, yet there are no radiologic or urologic guidelines defining urethral narrowing. This study takes the first step toward creating such guidelines by demonstrating intra- and inter-observer reliability among four highly specialized radiologists. The conditions in this study reflected how radiologists would read in real-life practice, using an established protocol [1] with all necessary voiding views and ensuring independent review to prevent reviewer bias. 

Despite these strengths, this study occurred at a single institution, without external validation from outside radiologists. It is unclear whether radiologists from diverse backgrounds would demonstrate this degree of inter-observer reliability. Given the frequent use of VCUGs at our institution for evaluating urethral narrowing, the degree of inter- and intra-observer reliability may be lower at institutions where radiologists have less experience in this application of VCUG. An additional limitation of this study is the lack of standardization in VCUG grading, reflected in the use of the “other” category by one grader to discuss multi-site or overlapping narrowing. Nonetheless, it is notable that inter- and intra-observer reliability remained high for location when agreement was assessed among the other three reviewers. These results suggest that establishing formalized grading criteria could further improve reader agreement. Other emerging techniques using artificial intelligence have already demonstrated promising results in VCUG grading, such as automating the differentiation between low- and high-grade vesicoureteral reflux (VUR) in pediatric populations [14]. While investigational, the success of machine learning with VUR detection highlights its potential as a tool for grading urethral narrowing on VCUG, potentially reducing inter-reader variability.

With this imaging reliability study completed, multicenter studies will be necessary to assess the generalizability of these initial findings and to evaluate the role of standardized grading criteria on VCUG interpretation. Future research should also investigate the relationship between urethral narrowing, as detected on VCUG, and clinically meaningful outcomes. 

## Conclusions

In summary, this single-institution study demonstrated high intra- and inter-observer reliability among radiologists for evaluating the presence, severity, and location of adult female urethral narrowing on VCUG. VCUG has been established as a safe and accessible method for assessing the female urethra, and these results specifically highlight its ability to measure urethral narrowing reliably. Future research will be necessary to validate these findings in a more extensive, multicenter study and to correlate VCUG imaging with clinical outcomes. Ultimately, the goal is to establish standardized numerical criteria for grading urethral narrowing on VCUG, potentially with the aid of artificial intelligence programs. These next steps are critical to improving diagnostic accuracy in the field of URPS.
